# Case Report: A good response to furmonertinib second-line treatment of an advanced lung adenocarcinoma patient with a rare EGFR exon 20 N771_P772insH mutation: A case report and literature review

**DOI:** 10.3389/fphar.2022.964606

**Published:** 2022-08-17

**Authors:** Xiao Zhang, Huan Han, Jiuzhou Zhao, Xiao Liu, Jianbo Zhang, Rui Sun, Shaomei Li, Baoxing Liu, Hui Zhu, Shuyue Jiao, Xiang Li, Hong Tang

**Affiliations:** ^1^ Department of Medical Oncology, The Affiliated Cancer Hospital of Zhengzhou University and Henan Cancer Hospital, Zhengzhou, China; ^2^ Department of Pathology, The Affiliated Cancer Hospital of Zhengzhou University and Henan Cancer Hospital, Zhengzhou, China; ^3^ Department of Radiotherapy, The Affiliated Cancer Hospital of Zhengzhou University and Henan Cancer Hospital, Zhengzhou, China; ^4^ Department of Thoracic Surgery, The Affiliated Cancer Hospital of Zhengzhou University and Henan Cancer Hospital, Zhengzhou, China

**Keywords:** NSCLC, EGFR exon 20 insertion mutation, N771_P772insH, furmonertinib, second-line

## Abstract

**Background:** Lung adenocarcinoma with the classical EGFR 19 deletion and exon 21 L858R point mutations has exhibited good responses to epidermal growth factor receptor tyrosine kinase inhibitors (EGFR-TKIs) treatment. However, the sensitivity of uncommon EGFR exon 20 insertion mutation to third-generation EGFR-TKIs has not been determined. Although emerging targeted therapies for EGFR exon 20 insertion mutation have been reported in recent years, such patients still have a poorer prognosis than those with typical or wild-type EGFR mutations.

**Case summary:** Here, we report the case of a 57-year-old man with advanced non-small cell lung cancer (NSCLC) with a rare EGFR exon 20 N771_P772insH mutation. The patient was treated with furmonertinib as second-line therapy. Although his pleural effusion was more than before that during treatment, various examination results showed that the pleural effusion was closely related to hypoproteinemia; thus, local progression was not considered. His cough was significantly alleviated, and the dose was well tolerated. The patient was evaluated for a remarkable progression-free survival (PFS) of 10.0 months, a duration of response (DOR) of 8.0 months, and an overall survival (OS) of 22.0 months, which had not previously been achieved.

**Conclusion:** The present study indicated that furmonertinib might be a good treatment option for first-line progressive NSCLC patients with EGFR exon 20 insertion mutation.

## Introduction

Lung cancer is the most frequent cancer and the leading cause of cancer-related death worldwide (Bray et al., 2018). Approximately 85% of lung cancer cases are non-small cell lung cancer (NSCLC) (Thai et al., 2021), of which lung adenocarcinoma is the most common subtype. Significantly, approximately 40–64% of Asian lung adenocarcinoma patients have epidermal growth factor receptor (EGFR) gene mutations (Midha et al., 2015). Exon 19 deletion and exon 21 L858R point mutations are common mutations in EGFR and have shown a favorable response to EGFR tyrosine kinase inhibitors (EGFR-TKIs) and good prognosis (Sasaki et al., 2007; Yasuda et al., 2013). However, the clinical implications of rare EGFR mutations remain unclear and have poor efficacy and prognosis. Notably, these mutations include exon 20 insertion mutation, accounting for 4%–12% of all EGFR mutations (Burnett et al., 2021). Current evidences suggested that first-line treatment with chemotherapy is superior to EGFR-TKIs, with better survival and response for exon 20 insertion mutation (Wu et al., 2019; Yang et al., 2020; Shah et al., 2022). However, chemotherapy is less favorable for subsequent treatment lines, and new targeted therapies could have unexpected effects (Burnett et al., 2021).

Amivantamab (JNJ-372) and mobocertinib (TAK-788) have already been approved as second-line therapies in patients with advanced NSCLC harboring EGFR exon 20 insertion mutation after progression to platinum-based chemotherapy (Markham, 2021; Syed, 2021). Poziotinib exhibited positive effects in NSCLC patients with EGFR exon 20 insertion mutations (Prelaj et al., 2021). However, the efficacy of these novel agents is not quite satisfactory. Recently, B. Han et al. has reported the significant efficacy of furmonertinib in NSCLC with EGFR exon 20 insertion mutation in a preclinical study (Han et al., 2021). In this case, we report a lung adenocarcinoma patient with a rare EGFR exon 20 insertion mutation (N771_P772insH) who responded well to furmonertinib (alflutinib, AST2818) after first-line chemotherapy progression, achieving a progression-free survival (PFS) of 10.0 months, a duration of response (DOR) of 8.0 months, and an overall survival (OS) of 22.0 months, which had not yet been achieved.

## Case report

**FIGURE 1:**
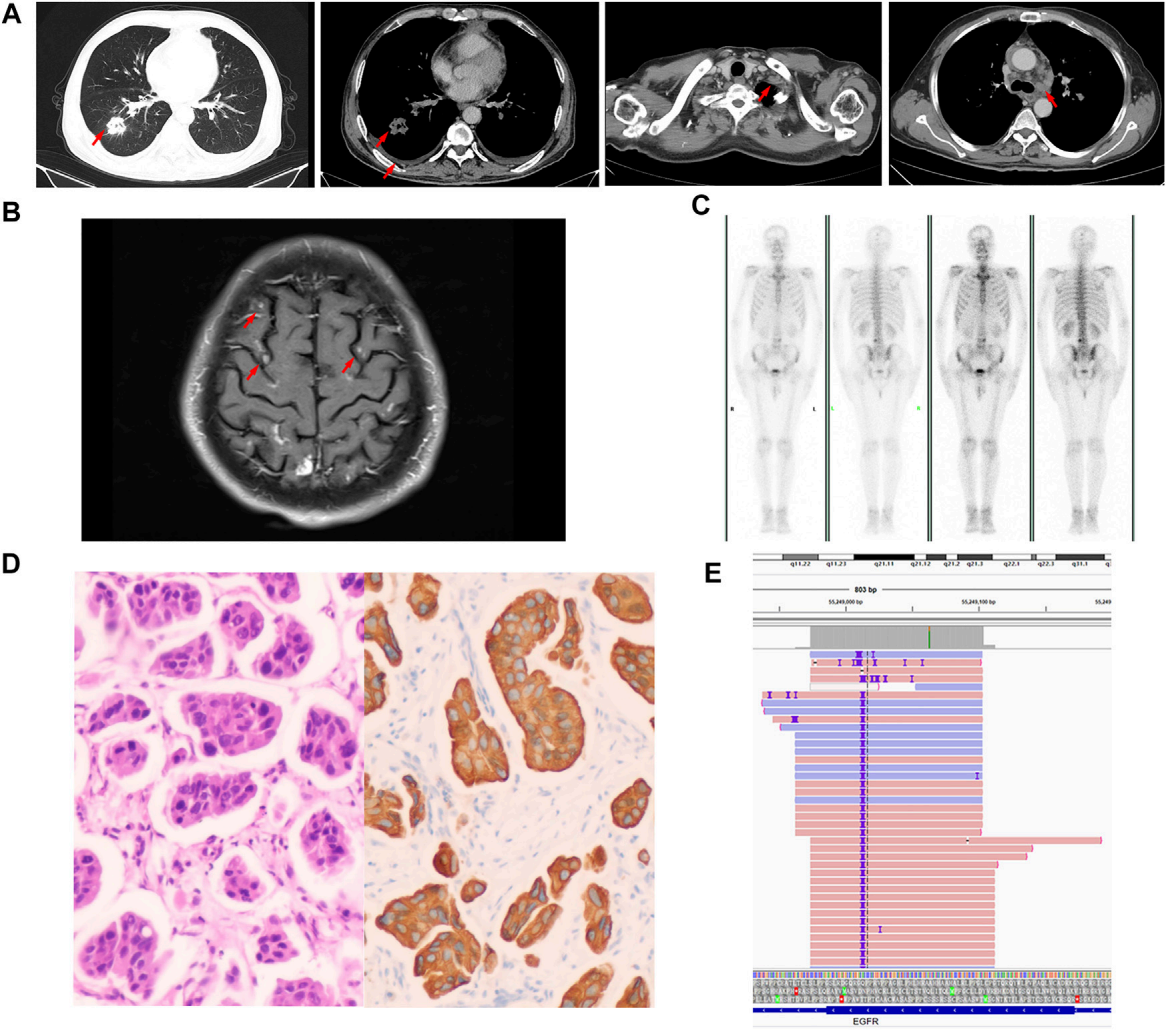
Baseline data. **(A)** Computed tomography (CT) images. **(B)** Magnetic resonance imaging (MRI). **(C)** Positron emission tomography-computed tomography (PET-CT) image. **(D)** Representative histopathological image of the tumor (H&E staining). **(E)** Next-generation sequencing showed a p.N771-P772insH (c.2311-2312insACC) mutation in EGFR exon 20.

On 15 August 2020, a 57-year-old man who had smoked an average of 15 cigarettes per day for more than 20 years presented at the lung cancer clinic of our hospital with a chronic cough and chest pain. The patient had no family history of tumors. Computed tomography (CT) revealed a 3.2 × 3.1 cm density mass in the lower right lung, multiple lymph node enlargements, bits of pleural effusion on both sides and right pleural metastasis (Figure 1A). Magnetic resonance imaging (MRI) showed scattered enhanced nodules in the brain, with metastasis considered (Figure 1B). Positron emission tomography-computed tomography (PET-CT) suggested active metabolism in the skull and fourth lumbar vertebra, with metastasis considered (Figure 1C). The patient was diagnosed with lung adenocarcinoma by ultrasound-guided lymph node biopsy of the left neck biopsy (Figure 1D). Next-generation sequencing (NGS) of the biopsy specimen indicated that a p.N771-P772insH (c.2311-2312insACC) mutation was in the EGFR gene, and the mutation abundance accounted for 61.1% (Figure 1E). According to the latest update of the Catalog of Somatic Mutations in Cancer (COSMIC) database (https://cancer.sanger.ac.uk/cosmic), the N771-P772insH mutation accounts for only 0.018% of EGFR mutations (5/27,066). Based on these data, the patient was diagnosed with stage IVB lung adenocarcinoma (cT2aN3M1c of TNM staging system).

**FIGURE 2:**
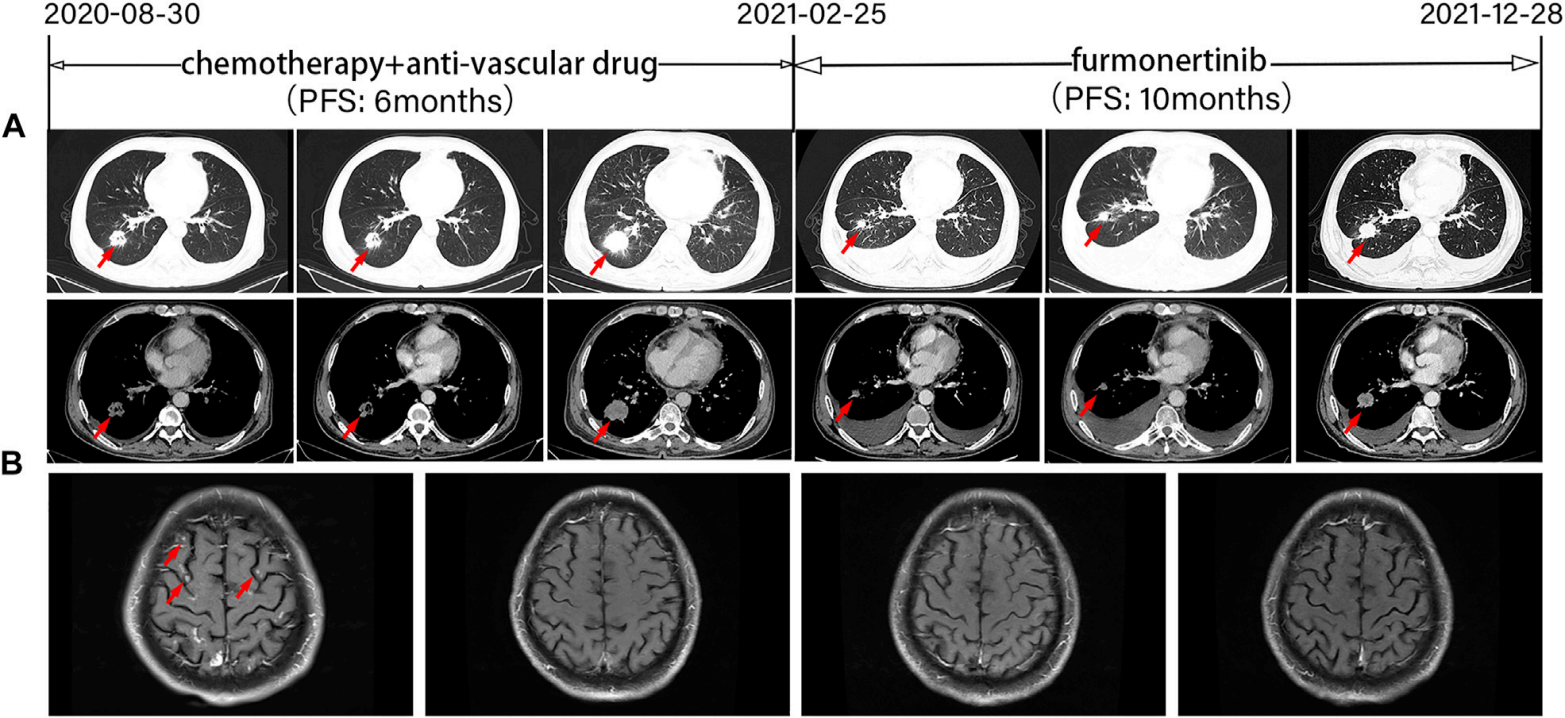
Tumor progression of the patient before and after treatment. **(A)** Representative computed tomography images at various points. CT images revealed lesions in the lower right lung. **(B)** Magnetic resonance images at various points. PFS, progression-free survival. The timeline of therapies, therapeutic regimens and tumor progression are indicated (top). The tumor is indicated by red arrows. SD, stable disease; PD, progressive disease; PR, partial response.

According to the relevant literatures (Lund-Iversen et al., 2012; Naidoo et al., 2015; Yang et al., 2015; Burnett et al., 2021), we initiated treatment with a platinum-containing two-drug chemotherapy combined with an anti-vascular drug (pemetrexed and carboplatin plus bevacizumab) after informed consent was obtained. The accompanying treatment plan consisted of zoledronic acid injection for osseous metastases. Based on the Response Evaluation Criteria in Solid Tumors (RECIST) 1.1 (Eisenhauer et al., 2009), stable disease (SD) was observed during treatment by chest CT (Figure 2A), and head MRI revealed PR (Figure 2B). The patient received 4 cycles of pemetrexed and carboplatin plus bevacizumab and 3 cycles of maintenance therapy with pemetrexed plus bevacizumab. However, 6 months later, the patient experienced increased chest tightness and coughing. CT showed an increased mass of solid components in the lower lobe of the right lung, increased multiple lymph nodes, and obviously thickened pleura on both sides (Figure 2A). The patient was diagnosed with progressed disease (PD) according to RECIST 1.1 (Eisenhauer et al., 2009). For personal reasons, he refused to undergo additional tissue biopsy or a liquid biopsy to assess circulating tumor DNA (ctDNA). Amivantamab and mobocertinib were not available on the Chinese market. The patient was then treated with furmonertinib (160 mg/day) with informed consent. This treatment was approved by the Ethics Committee of Henan Cancer Hospital. Two months later, a CT scan showed a significant reduction in the right lung lower lobe mass, while the pleural effusion on both sides increased. Because the patient had low albuminemia, no tumor cells were detected in the pleural effusion combined with pleural effusion routine and biochemical examination results tended to transudate, we speculated that the nature of pleural effusion was transudate. Therefore, the efficacy evaluation was partial response (PR) according to RECIST 1.1 (Eisenhauer et al., 2009) (Figure 2A). MRI indicated that the patient’s brain metastases (BMSs) were constantly stable (Figure 2B). Disease developed again, as shown by a CT scan (Figure 2A) in December 2021. The PFS was 10.0 months, and the DOR was 8.0 months. Subsequently, the patient was treated with chemotherapy regimens. The last follow-up was 30 June 2022. The OS was 22.0 months (not yet achieved). During furmonertinib treatment, no severe adverse effects (AEs) were observed in this process except for transaminase elevation and low albuminemia (grade II).

## Discussion

Many studies have reported the clinical efficacy of different first-line treatment regimens in patients with exon 20 insertion mutation. There is no doubt that platinum and pemetrexed-based chemotherapy is currently the most effective first-line treatment for EGFR exon 20 insertion patients (Lund-Iversen et al., 2012; Naidoo et al., 2015; Yang et al., 2015; Burnett et al., 2021). However, there are still no satisfactory drugs for subsequent treatment routes in China. Here, we adopted furmonertinib (160 mg qd po) targeted treatment after the patient’s first-line progression. The patient achieved 10.0 months of PFS, 8.0 months of DOR and 22.0 months or more of OS, constituting an exciting result.

It is important to note that patients with exon 20 insertion mutation have a poorer prognosis than those with typical or wild-type EGFR mutations in a variety of standard therapies (Xu et al., 2016; Burnett et al., 2021). In almost all studies, the median OS time of patients with classical mutations was twice that of those with exon 20 insertion mutations (17.3–31.6 months vs. 4.8–16.8 months) (Burnett et al., 2021). Epidemiological analysis showed that the clinical and pathological characteristics of EGFR exon 20 insertion mutation patients were similar to those of classical EGFR mutation patients, and most of these patients were Asian, female, nonsmoking, and elderly and had adenocarcinoma (Burnett et al., 2021). However, the patient in our case was a smoker and male, which could be related to molecular heterogeneity. A multicenter, observational study showed that smokers had a worse prognosis than nonsmokers (OS: 12 months versus 21 months; HR: 0.27; 95% CI: 0.08–0.87; *p* = 0.03) (Beau-Faller et al., 2014).

**FIGURE 3:**
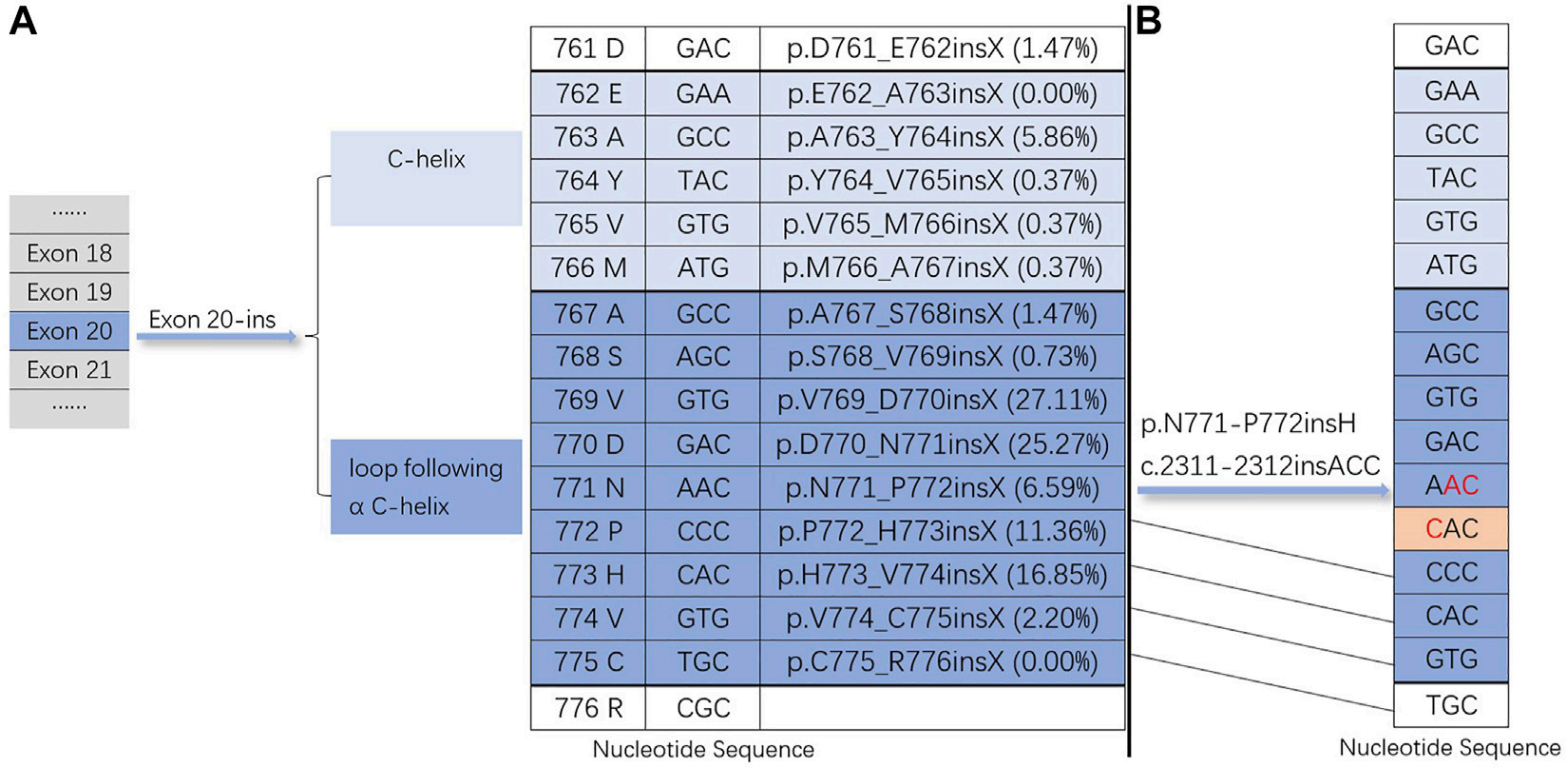
Exon 20 insertion mutations. **(A)** The insertion site of the EGFR exon 20-ins mutation and the insertion mutation frequency of EGFR exon 20 at different locations based on the COSMIC database (https://cancer.sanger.ac.u1c/cosmic). **(B)** Nucleotide sequence of our case (EGFR exon 20-ins: p.N771-P772insH c.2311-2312insACC).

EGFR exon 20 insertion mutation can promote the activation of the EGFR kinase domain, affecting ATP and leading to carcinogenesis (Lai et al., 2013). EGFR exon 20 contains nucleotides translated into amino acids at positions 762–823. They include an α C-helix (G762-M766) and a loop following the α C-helix (A767-C775) (Yasuda et al., 2012; Yasuda et al., 2013) (Figure 3). Based on the COSMIC database (https://cancer.sanger.ac.uk/cosmic), we calculated the insertion mutation frequency of EGFR exon 20 at different locations (Figure 3). Exon 20 insertion is preferentially located behind the C-helix (A767 to C775) and promotes the activation of the EGFR kinase domain, leading to tumorigenesis, but it does not increase affinity for EGFR tyrosine kinase inhibitors (Yasuda et al., 2012; Lai et al., 2013). Notably, our case showed that exon 20 insertion was located behind the C-helix (N771-P772) but still exhibited a good affinity for EGFR-TKIs (Figure 3). The associated bypass pathways (EGFR ECD mutations, PIK3CA mutations and so on) activated by EGFR exon 20 insertion mutation are closely associated with primary resistance to first-generation TKIs (Lai et al., 2013).

Except for EGFR A763_Y764insFQEA and a few other subtypes (Yasuda et al., 2013; Naidoo et al., 2015), most EGFR exon 20 insertion mutations can affect the affinity of the EGFR receptor for reversible inhibitors, resulting in resistance to first-generation EGFR-TKIs with poor efficacy (Yasuda et al., 2012; Yasuda et al., 2013; Lei et al., 2020). First-generation EGFR-TKIs (erlotinib, gefitinib and icotinib) therapy is less effective in patients with EGFR exon 20 insertion mutation (mPFS: 2.00 months [95% CI, 0.00–5.41 months]) (Xu et al., 2016). Many studies have shown that some second- or third-generation EGFR-TKIs have moderate effects on patients with EGFR exon 20 insertion mutation. The clinical benefit of afatinib was lower in patients with exon 20 insertion mutation [mPFS: 2.7 months (range: 1.8–4.2); mOS: 9.2 months (range: 4.1–14.2)] (Yang et al., 2015). A phase I/II study revealed that osimertinib (80 mg qd po) had limited efficacy in NSCLC patients with EGFR exon 20 insertion mutation (mPFS: 3.8 months; mOS: 15.8 months). Of the twelve evaluated patients, none experienced CR/PR, 7 experienced SD (58.3%), and 5 experienced PD (41.7%) (Yasuda et al., 2021), while the higher dose of osimertinib might have potential efficacy. Recently, Floc’h et al. have showed significant antitumor activity of both osimertinib and its circulating metabolite AZ5104 against NSCLC harboring an EGFR exon 20 insertion mutation using CRISPR-Cas 9 engineered cell lines. Osimertinib and AZ5104 inhibit signal pathways and cellular growth in EGFR exon 20 insertion mutant cell lines *in vitro* (Floc’h et al., 2018). These information support clinical testing the higher dose of osimertinib in patients with EGFR exon 20 insertion mutant NSCLC.

The FDA recently approved two targeted breakthrough therapies, amivantamab [JNJ-372] and mobocertinib [TAK-788], which were designated for the treatment of metastatic NSCLC patients with EGFR exon 20 insertion mutation who have progressed during or after platinum chemotherapy (Markham, 2021; Syed, 2021). In the CHRYSALIS phase I study (NCT02609776), forty NSCLC patients with EGFR exon 20 insertion mutation were treated with amivantamab, an EGFR-MET bispecific antibody. The median progression-free survival was 8.3 months (95% CI, 6.5–10.9) (Park et al., 2021). The phase I/II open-label nonrandomized clinical trial (NCT02716116) evaluated the treatment outcomes of mobocertinib in patients with previously treated EGFR exon 20 insertion NSCLC. The platinum-pretreated patients (PPP) cohort confirmed a median duration of response of 17.5 months (95% CI, 7.4–20.3), a median progression-free survival of 7.3 months (95% CI, 5.5–9.2) and a median overall survival of 24.0 months (95% CI, 14.6–28.8) (Zhou et al., 2021). The ZENITH20-2 trial (NCT03318939.) demonstrated antitumor activity of poziotinib in previously treated patients with EGFR exon 20 insertion mutation advanced NSCLC [mPFS: 5.5 months (95% CI, 3.9 to 5.8); mDOR: 5.1 months (95% CI, 4.2 to 5.5)] (Le et al., 2022). For advanced NSCLC patients with EGFR exon 20 insertion mutations, CLN-081 (TAS6417) had a controlled safety profile and positive antitumor activity in the experimental dose range at European Society for Medical Oncology (ESMO) 2020 and American Society of Clinical Oncology (ASCO) 2022 (Piotrowska1 et al., 2020; Yu et al., 2022). Two ongoing phase 1/2 studies (WK-KONG1, NCT03974022 and WU-KONG2, CTR20192097) indicate that sunvozertinib has comparable efficacy and safety profiles in platinum-pretreated patients with EGFR exon 20 insertion mutations, irrespective of prior anti-PD(L)1 treatment at ASCO 2022 (Pasi et al., 2022). These compounds are emerging drugs for NSCLC patients with EGFR exon 20 insertion mutations.

Furmonertinib (alflutinib, AST2818), a structural analog of osimertinib, is a new third-generation irreversible and selective EGFR-TKI developed by Shanghai Allist Pharmaceuticals Co., Ltd. (Deeks, 2021; Meng et al., 2022). Furmonertinib was approved in China on 3 March 2021, for the treatment of NSCLC patients with EGFR T790M mutation who have progressed during or after EGFR-TKIs therapy (Deeks, 2021). Furmonertinib has a wider safety window than osimertinib due to the introduction of a unique trifluoroetoxypyridine structure, which is highly hydrophobic. The hollow hydrophobic pocket, composed of hydrophobic amino acids L792 and M793, has a high affinity for the binding region of EGFR ATP (Shi et al., 2020). Furmonertinib is metabolized primarily by cytochrome P450 3A4 (CYP3A4), and it is a potent inducer of CYP3A4 (Wu et al., 2022). Over 95% of furmonertinib and its metabolites are covalently bound to plasma proteins, and the main forms of free drugs are the parent drug and AST5902, both of which have similar pharmacological activities *in vivo* (Meng et al., 2022). Both furmonertinib and its main metabolite, AST5902, have high antitumor activity and are highly selective, and both can penetrate the blood‒brain barrier (Shi et al., 2020; Meng et al., 2022; Wu et al., 2022). Furmonertinib is mainly distributed in the lung within 4 h after administration. Therefore, furmonertinib might increase lung cancer treatment efficacy (Meng et al., 2022).

The phase III FURLONG trial reported that advanced NSCLC patients with EGFR-sensitive mutations treated with furmonertinib had a significantly longer median progression-free survival than those treated with gefitinib [mPFS: 20.8 vs. 11.1 months, HR: 0.44 (95% CI: 0.34–0.58), *p* < 0.0001], and the extension was 9.7 months at European Lung Cancer Congress (ELCC) 2022 (Shi1 et al., 2022). The FAVOUR 1 study (Phase Ib study, NCT04858958) demonstrated that furmonertinib effectively inhibited the expression of EGFR 20 insertion mutation in BaF3 cells in a preclinical evaluation (Han et al., 2021). The preliminary results of the FAVOUR study showed that all 10 EGFR exon 20 insertion mutant advanced NSCLC patients in Group 1 who were initially treated with furmonertinib 240 mg qd showed tumor shrinkage in target lesions (median best percent change, -43.0% [-72.3%, -3.0%]) (Han et al., 2021). Groups 2 and 3 received furmonertinib (240 mg qd and 160 mg qd, respectively) as a second-line treatment, and the major results are pending (Han et al., 2021). The common AEs during treatment were diarrhea, paronychia, skin fissures etc. No grade≥3 AEs were observed. A study reported at ASCO 2022 showed that furmonertinib at twice the conventional dose achieved a good curative effect in 15 NSCLC patients with EGFR exon 20 insertion mutations (ORR: 53.5%; DCR: 100%) (XiaoyueZhou et al., 2022). A phase I, multicenter clinical study (NCT04958967) is exploring the efficacy and safety of furmonertinib at different doses (160 mg/day and 240 mg/day) in locally advanced or metastatic NSCLC patients with EGFR exon 20 insertion mutations (ClinicalTrials.gov, 2021). The primary endpoint was the overall response rate in this study. As a novel third-generation EGFR-TKI, furmonertinib has shown promising antitumor activity in EGFR exon 20 insertion NSCLC patients, bringing new light to targeted therapies for EGFR exon 20 insertion NSCLC patients. Furmonertinib is a good choice for improving the prognosis and curative effects in EGFR exon 20 insertion patients with lung adenocarcinoma.

The limitations of this case are obvious. The lack of biopsies prevented further biomarker analysis after first-line treatment progression.

## Conclusion

In this case report, the patient received furmonertinib as a second-line therapy and achieved a PFS of 10.0 months, a DOR of 8.0 months, and an OS of 22.0 months (not yet achieved). The sites, sequences and lengths of EGFR exon 20 insertion are diverse. The treatment of the N771-P772insH variant of the EGFR exon 20 insertion mutation has not been previously reported, and this report is the first clinically significant case of treatment of the related subtype, with encouraging results. Successful treatment with furmonertinib could provide a new treatment option for this subtype of exon 20 insertion mutation, especially for patients with first-line chemotherapy progression. Further studies are required to validate the efficacy of furmonertinib in these patients, although data from case reports such as this one provided some supportive evidence.

## Data Availability

The original contributions presented in the study are included in the article/supplementary material, further inquiries can be directed to the corresponding author.
